# Largely Enhanced Thermoelectric Power Factor of Flexible Cu_2−x_S Film by Doping Mn

**DOI:** 10.3390/ma16227159

**Published:** 2023-11-14

**Authors:** Xinru Zuo, Xiaowen Han, Yiming Lu, Ying Liu, Zixing Wang, Jiajia Li, Kefeng Cai

**Affiliations:** Key Laboratory of Advanced Civil Engineering Materials of Ministry of Education, Shanghai Key Laboratory of Development and Application for Metal-Functional Materials, School of Materials Science & Engineering, Tongji University, Shanghai 201804, China; 2132856@tongji.edu.cn (X.Z.); 18616350526@163.com (X.H.); 1651650@tongji.edu.cn (Y.L.); liuying_polymer@163.com (Y.L.); wzxing2023@163.com (Z.W.); sduljj2018@163.com (J.L.)

**Keywords:** Cu_2_S, thermoelectric, hydrothermal synthesis, doping, flexible film

## Abstract

Copper-sulfide-based materials have attracted noteworthy attention as thermoelectric materials due to rich elemental reserves, non-toxicity, low thermal conductivity, and adjustable electrical properties. However, research on the flexible thermoelectrics of copper sulfide has not yet been reported. In this work, we developed a facile method to prepare flexible Mn-doped Cu_2−x_S films on nylon membranes. First, nano to submicron powders with nominal compositions of Cu_2−x_Mn_y_S (y = 0, 0.01, 0.03, 0.05, 0.07) were synthesized by a hydrothermal method. Then, the powders were vacuum-filtrated on nylon membranes and finally hot-pressed. Phase composition and microstructure analysis revealed that the films contained both Cu_2_S and Cu_1.96_S, and the size of the grains was ~20–300 nm. By Mn doping, there was an increase in carrier concentration and mobility, and ultimately, the electrical properties of Cu_2−x_S were improved. Eventually, the Cu_2−x_Mn_0.05_S film showed a maximum power factor of 113.3 μW m^−1^ K^−2^ and good flexibility at room temperature. Moreover, an assembled four-leg flexible thermoelectric generator produced a maximum power of 249.48 nW (corresponding power density ~1.23 W m^−2^) at a temperature difference of 30.1 K, and had good potential for powering low-power-consumption wearable electronics.

## 1. Introduction

Thermoelectric (TE) materials can directly convert heat into electricity and vice versa. This has attracted great attention for power generation and cooling [1]. With the growing demand for electrical appliances, there is an urge to produce renewable sources using flexible, eco-friendly, and economical materials. Flexible TE films (f-TEFs), which can fulfill this need, can generate electricity by utilizing the temperature difference (Δ*T*) between the human body and the environment [2]. The TE performance of a material is determined by the dimensionless figure of merit *ZT*, defined as *ZT = α*^2^*σT/κ*, where *α* is the Seebeck coefficient, *σ* is the electrical conductivity, *κ* is the thermal conductivity, *T* is the absolute temperature, and α^2^σ is called power factor (PF) that is related to electrical properties [3].

In recent years, substantial efforts have been mainly devoted to free-standing films [4,5,6] and films on flexible substrates [7,8,9,10,11,12]. For free-standing films, conductive polymers and their based composites have been widely studied due to their excellent flexibility and low thermal conductivity [13,14]. However, their *ZT* values still cannot be compared to those of inorganic TE materials. Inorganic films on flexible substrates can combine the flexibility of the substrate (such as polyimide, nylon, or paper) with the high *ZT* of inorganic TE materials, effectively balancing flexibility and TE performance [15]. For instance, a flexible p-type Bi_0.4_Sb_1.6_Te_3_/Te composite film prepared on a Kapton surface by screen printing combined with pressure-less sintering (450 °C, 45 min) achieved a reasonably high PF value of ~3000 μW m^−1^ K^−2^ (*ZT* ~ 1) at 300 K [9]. It showed a 3% increase in resistance after 1000 times bending around a 10 mm-radius rod. In 2019, our group developed a facile method to fabricate flexible Ag_2_Se films on nylon membranes [7]: Se nanowires (NWs) were synthesized by a wet chemical method and used as templates to prepare Ag_2_Se NWs, and Ag_2_Se film was formed on porous nylon membranes by vacuum filtration and then hot-pressing (HP). The Ag_2_Se films exhibited excellent flexibility and a PF of ~987 μW m ^−1^ K^−2^ (*ZT* ~ 0.5) at 300 K. Since then, we have worked to improve the PF [16,17,18]. For example, polypyrrole (PPy) was in situ polymerized to fabricate Ag_2_Se/Se/PPy composite films on nylon membranes [17], and a PF of ~2240 μW m^−1^ K^−2^ at 300 K was achieved. Recently, Lei et al. [19] prepared Ag_2_Se films by immersing Ag films sputtered on polyimide substrates into Se/Na_2_S solution for selenization, and the PF at room temperature (RT) was ~2590 ± 414 μW m^−1^ K^−2^. Despite the excellent TE properties of Bi_2_Te_3_ and Ag_2_Se at RT, the elements Te and Se are not abundant and are toxic. So, for practical TE applications, it was necessary to search for low-cost inorganic materials without toxic elements.

Copper sulfide (Cu_2−x_S, 0 ≤ x ≤ 0.25), a typical liquid-like semiconductor [20], which has low lattice thermal conductivity [21], has been considered to be the candidate material to decouple electrical and thermal properties. Meanwhile, the elements Cu and S are abundant, cheap, and nontoxic; thus, Cu_2−x_S is an economical and environmentally friendly TE material [22,23]. It forms a series of compounds ranging from copper-rich to copper-deficient, such as Cu_2_S, Cu_1.96_S, Cu_1.92_S, and Cu_1.8_S, whose crystal structures and TE performances vary with copper content. Typically, Cu_2_S possesses a comparatively high *α* of ~300 μV K^−1^ and poor *σ* (≤10 S cm^−1^) at RT [24]. The crystal structure of Cu_2_S undergoes complex change with increasing temperature: it is in a monoclinic-chalcocite phase at RT, which transforms into a hexagonal phase above 370 K and then into a cubic phase near 709 K [9,25]. The current research on copper-sulfide bulks mainly focuses on the medium and high-temperature regions. For example, Tang et al. [26] introduced a 3D graphene heterointerface into the Cu_2−x_S matrix by mechanical alloying and spark plasma sintering (SPS) and obtained a high PF of 1197 μW m^−1^ K^−2^ (*ZT* ~ 1.56) at 873 K. And Cu_2_S bulk incorporated Ag nanoparticles prepared by a hydrothermal method realized a high PF of 1698 μW m^−1^ K^−2^ (*ZT* ~ 1.4) at 773 K [27]. Recently, Li et al. [28] synthesized Cu_2_S-Cu_1.96_S phase junctions by retaining surface 1-dodeca-nethiol (DDT) ligands and pushed the peak *ZT* value to 2.1 at 932 K. However, bulk materials are usually costly and not suitable for flexible TE devices [29,30].

To date, there has been little attempt at flexible copper-sulfide-based films. For example, Cu_2_S/poly (3,4-ethylene dioxythiophene):poly (styrene sulfonate) (PEDOT:PSS) hybrid films were fabricated by screen printing on polyimide. And when the content ratio of Cu_2_S/PEDOT:PSS was 1:1.2, the prepared film showed a maximum PF of 20.60 µW m^−1^ K^−1^ at ~400 K [31]. Self-supporting and flexible Cu_2_S/PEDOT:PSS composite TE films were prepared by a vacuum filtration method, and the maximum PF was 56.15 μW m^−1^ K^−2^ at 393 K [32]. Both of these films had good flexibility, but the TE performances were low.

Recently, our group [33] reported a one-pot method for the synthesis of Ag_2_Se powders combined with vacuum filtration and HP to prepare flexible Ag_2_Se film on a nylon membrane, which possessed a PF of ~2042 μW m^−1^ K^−2^ at RT. This showed that flexible films can be prepared by the above method as long as the size of the powders is beyond the pore size of the nylon membrane. Herein, we designed a green and facile hydrothermal synthesis method to synthesize Cu_2−x_S powders without any corrosive chemicals and then prepared the Cu_2−x_S film on nylon membranes by vacuum filtration and then HP. As mentioned above, the *σ* of Cu_2_S is, unfortunately, low. There have been many strategies to improve the σ of TE materials, such as doping to tune carrier concentration (*n*) [27], introducing a second phase with high *σ* [23], and band engineering [34]. Copper ions in Cu_2_S are mobile ions, and doping positive ions with higher electronegativity at the copper site will increase the *n* by forming a stronger bond with sulfur and is a good strategy to optimize the electrical properties of Cu_2_S [35].

In this work, to improve the PF of the Cu_2−x_S film, we chose Mn as the dopant to enhance the *σ* while maintaining the *α*. Through the doping of Mn, an optimal film exhibited a PF of 113.3 μW m^−1^ K^−2^ at RT, and the output performance of an assembled flexible TE generator (f-TEG) was studied.

## 2. Experimental Section

### 2.1. Sample Synthesis

Mn-doped Cu_2−x_S powders were synthesized by a hydrothermal method, which was a modified method for Cu_2−x_S [36]. Typically, 3.6 mmol thiourea (Tu), 7.56 mmol CuCl_2_·2H_2_O, and MnCl_2_·4H_2_O were dissolved into 20 mL of deionized (DI) water. The nominal doping content of Mn in Cu_2−x_S was 0, 1, 3, 5, and 7% by molar ratio, marked as Cu_2−x_Mn_y_S (y = 0, 0.01, 0.03, 0.05, and 0.07). Afterward, they were mixed and sonicated for 30 min and then transferred into a 100 mL Teflon-lined stainless-steel autoclave at 180 °C for 18 h with a heating rate of 2 °C min^−1^. After cooling naturally, the products were washed with DI water and ethanol 3 times. The corresponding films were prepared by vacuum-assisted filtration of the powder dispersions on porous nylon membranes, then dried at 60 °C in vacuum overnight, and, finally, hot-pressed at 270 °C and 1 MPa for 30 min. The schematic diagram of the preparation process of the Cu_2−x_Mn_y_S film is shown in Figure S1 (see Supplementary Materials).

### 2.2. Assembly of the f-TEG

The Cu_2−x_Mn_0.05_S film was cut into four strips of 2 cm × 0.5 cm. Two ends of each strip were coated with a thin layer of Au by evaporation to reduce contact resistance. Then, the four strips were pasted on a polyimide substrate at intervals of 0.5 cm. Finally, Ag paste (SPI#04998-AB) was used to connect the strips in series.

### 2.3. Characterization and Property Measurement

X-ray diffraction (XRD, Bruker D8 Advance, Cu K_α_ radiation, Bruker, Shanghai, China) was performed. Scanning electron microscopy (SEM, Nova NanoSEM 450, Thermo Fisher Scientific, Shanghai, China) and energy-dispersive X-ray spectroscopy (EDS) were used to observe the surface and cross-sectional morphology and composition. X-ray photoelectron spectroscopy (XPS, ESALAB 250Xi Spectrometer Microprobe, Thermo Fisher Scientific, Shanghai, China) was used to analyze the composition and valence states of surface elements. The binding energy was calibrated by setting the standard value of C1s to 284.8 eV. Powders scraped from the Cu_2−x_Mn_0.05_S film were observed by transmission electron microscopy (TEM, JEM-2100F, JEOL, Shanghai, China). The films were cut into 1.3 cm × 0.3 cm strips to test the temperature dependence of σ and α with a TE test system (CTA Cryoall, Beijing Cryoall, Shanghai, China) under the protection of He. The Hall carrier concentration (*n_H_*) and mobility (*μ_H_*) were measured using the Van der Pauw method with a Hall effect measurement system (HMS-7000, Ecopia, Shanghai, China). To test flexibility, σ was measured before and after bending around a 4 mm-radius rod to test flexibility.

The assembled f-TEG was connected with wires into a circuit, as shown in Figure S2 (see Supplementary Materials) for the output performance test [37,38]. The hot-end temperature was controlled by heating a copper block (*T* + Δ*T*) and the other end was put on an adiabatic foam acting as the cold side (*T*). The temperature at both ends was measured by two thermocouples. The output voltage and current were collected by adjusting the variable resistor box at a specific Δ*T*, which was varied by setting different heating temperatures.

## 3. Results and Discussion

Figure S3 (see Supplementary Materials) shows XRD patterns of the powders. The main phase of undoped copper sulfide can be indexed to tetragonal Cu_2_S (PDF No. 72-1071) with evident diffraction peaks at 2θ = 31.6°, 32.6°, 39.0°, 39.8°, 45.3°,46.0°, and 48.2°, which correspond to (110), (111), (104), (113), (200), (201), and (202) planes of Cu_2_S, respectively. There is no obvious shift of XRD peaks and no manganese-related compounds are detected. There are very weak diffraction peaks in Figure S3 when y = 0.07, which belong to digenite Cu_1.8_S (PDF No. 24-0061). According to [21,36], the related reactions are proposed as follows:

(1) $${\mathrm{NH}}_{2}{\mathrm{CSNH}}_{2}+3H_{2}O\;\to H_{2}S+2{NH_{4}}^{+}{+\mathrm{CO}}_{3}^{2-}$$

(2) $$H_{2}S+{Cu^{2}}^{+}\to \mathrm{CuS}+2H^{+}$$

(3) $$\mathrm{CuS}\overset{∆}{\to }{\mathrm{Cu}}_{2-x}S$$

**Figure 1:**
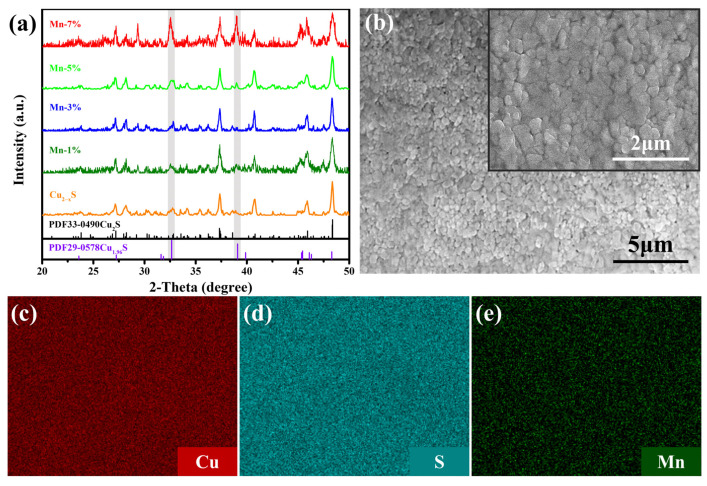
(**a**) XRD patterns of the Cu_2−x_Mn_y_S films with varying doping content of Mn. (**b**) A typical SEM image at low magnification, inset at high magnification. (**c**–**e**) Corresponding element mappings of the Cu_2−x_Mn_0.05_S film.

Hydrogen sulfide comes from the decomposition of thiourea in the early stage of the reaction and reacts with copper ions to produce CuS [36], which subsequently transforms into Cu_2−x_S during the heating process [21]. Figure 1a depicts the XRD patterns for the Cu_2−x_Mn_y_S films. It can be observed that the main diffraction peaks can be well indexed to monoclinic Cu_2_S (PDF No. 33-0490). In addition, two peaks at 2θ ~ 32.6° and 39.0° are detected, corresponding to (103) and (104) planes of tetragonal Cu_1.96_S (PDF No. 29-0578). Hence, the as-prepared films are composed of Cu_2_S with a small amount of Cu_1.96_S. When the nominal content of Mn is 7%, the XRD peaks for Cu_2_S broaden and the peaks for Cu_1.96_S become stronger.

Figure S4 (see Supplementary Materials) shows SEM images of Cu_2−x_Mn_y_S (y = 0.01, 0.03, 0.05, and 0.07) powders, with most particles being ~20–300 nm. A representative SEM image of the Cu_2−x_Mn_0.05_S film is shown in Figure 1b (the other films show similar morphology, see Supplementary Figure S5). The size of grains is <~300 nm. It can be seen from Figure 1b that the film is not very dense (as the sintering temperature was limited by the melting point of the nylon substrate). EDS results (Figure 1c–e) indicate that the elements of Cu, S, and Mn are homogeneously distributed in the Cu_2−x_Mn_0.05_S film.

**Figure 2:**
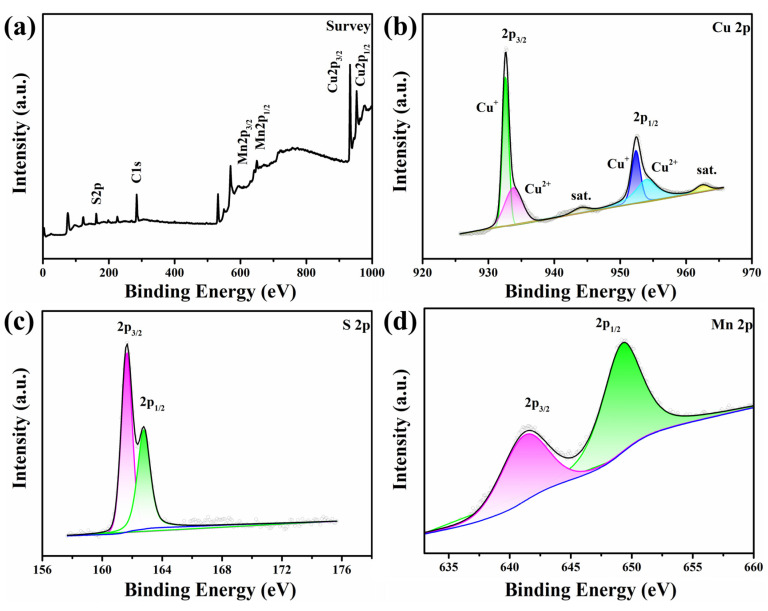
XPS spectra of the Cu_2−x_Mn_0.05_S film. (**a**) Survey scan. (**b**–**d**) High-resolution scans for Cu 2p, S 2p, and Mn 2p, respectively.

Figure 2a shows the XPS survey spectra of the Cu_2−x_Mn_0.05_S film; the signals of Cu, S, Mn, and C are detected. Two strong peaks at 932.6 eV (Cu 2p_3/2_) and 952.4 eV (Cu 2p_1/2_) (see Figure 2b) correspond to Cu^+^ [39]. The weak split peaks at about 933.8 and 954.0 eV are attributed to Cu^2+^, and the two satellite peaks located at 944.0 and 962.5 eV also correspond to Cu^2+^ [27,35], which proves the existence of Cu_1.96_S [40,41]. We estimated the ratio of Cu^+^:Cu^2+^ to be about 4.69:1, which is close to the value estimated by semi-quantitative analysis from the XRD result (5.11:1). Compared with the spectra of Cu_2−x_Mn_0.05_S powders (see Supplementary Figure S6), the ratio Cu^+^:Cu^2+^ becomes higher, indicating the partial conversion of Cu_1.96_S to Cu_2_S during HP. In Figure 2c, the characteristic peaks of S 2p_3/2_ and 2p_1/2_ are located at 161.6 and 162.8 eV, respectively, and the energy difference between them is about 1.2 eV, indicating the existence of S^2−^ [42,43,44]. Besides, the small resolved peaks with binding energies of 641.2 and 649.2 eV are Mn 2p_3/2_ and 2p_1/2_, respectively (see Figure 2d). The peak at 641.2 eV of Mn 2p_3/2_ is consistent with the previously reported value of MnS, which is attributed to the Mn-S bond [45]. The XPS and EDS results demonstrate the successful incorporation of Mn into copper sulfide. Mn has an initial valence of +2 and no strong oxidant is present in the reaction, so it is considered that Mn exists in the samples in a divalent state. In addition, because Cu^+^ (0.096 nm) and Cu^2+^ (0.072 nm) coexist in the Cu_2−x_S, while the ionic radius of Mn^2+^ (0.080 nm) is between them, the XRD peaks in Figure S3 and Figure 1a have no obvious shift.

**Figure 3:**
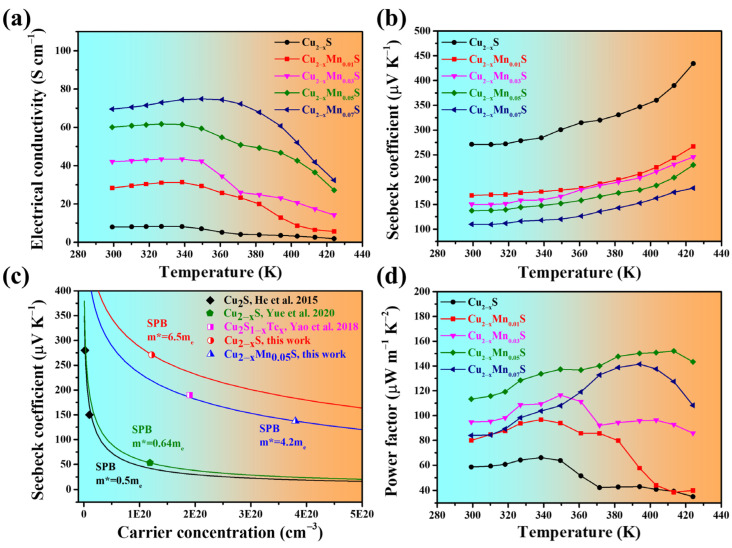
The temperature-dependent (**a**) electrical conductivities and (**b**) Seebeck coefficients. (**c**) Seebeck coefficient as a function of carrier concentration for Cu_2−x_S-, Cu_2−x_Mn_0.05_S-, and reported Cu_2−x_S-based bulks [21,50,51] at 300 K. (**d**) Temperature-dependent power factors of the Cu_2−x_Mn_y_S films.

Figure 3a,b,d show the temperature dependence of TE parameters of the Cu_2−x_Mn_y_S films. In the initial stage of temperature rise, the *σ* increases concomitantly, exhibiting a typical semiconductor behavior, and then it decreases when the temperature is near 340 K, which is lower than the transition temperature (*T_t_*) of Cu_2_S (370 K). The leftward shift of the *T_t_* is due to the presence of Cu_1.96_S, which undergoes a phase transition at 336 K [46]. The undoped Cu_2−x_S film possessed a high *α* of 271 μV K^−1^ and a low *σ* of 8 S cm^−1^ at RT. As the Mn doping amount increased, the *σ* increased, reaching ~70 S cm^−1^ when y = 0.07, nearly nine times as high as that of the undoped Cu_2−x_S film. Cu_2−x_S is a type of p-type semiconductor (see hereinafter). Manganese was doped into copper sulfide in the form of divalent ions, according to the XPS analysis, which should provide additional electrons in the Cu_2−x_Mn_y_S films to act as a donor. However, the *σ* at RT exhibited an opposite trend. This phenomenon is consistent with the change in *σ* of Mn- and Sn-alloyed Cu_2_S bulk obtained by the melting method [39]. Besides, through molecular orbital theory analysis, Wang et al. [47] revealed that the 3d orbital energies of Mn were similar to those of S 3p orbitals, causing the S 3p orbital to move away from the Cu-S bond, thereby weakening the Cu-S bond. Hence, the introduction of Mn weakens the Cu-S chemical bond, resulting in the formation of more Cu vacancies in the crystal lattice [39,48,49], namely, increasing the hole concentrations, which agrees with the Hall test result: At RT, the *n_H_* increased from 1.22 × 10^20^ for the Cu_2−x_S and to 3.80 × 10^20^ cm^−3^ for the Cu_2−x_Mn_0.05_S. In addition, the *μ_H_* also increased from 2.91 for the Cu_2−x_S to 4.37 cm^2^ V^−1^ s^−1^ for the Cu_2−x_Mn_0.05_S. Since the σ is proportional to the *n* and mobility (*μ*), defined as:

(4) σ=nqμ 

where *q* is the electron charge, the *σ* of the Cu_2−x_Mn_0.05_S film was ultimately improved.

The *α* increased with rising temperature (Figure 3b). And the positive *α* values also indicate that holes were the dominant charge carriers. As the Mn doping amount increased, the *α* decreased from 271 to 109.9 μV K^−1^ at RT, which is opposite to the trend of *σ* with the amount of doping. The *α* is proportional to *n*^−2/3^, expressed as follows:

(5) $$\alpha =\frac{8\pi^{2k_{B}^{2}}}{3eh^{2}}m^{*}T{\left(\frac{\pi }{3n}\right)}^{2/3}$$

where *k_B_* is the Boltzmann constant, *h* is the Planck constant, and m* is the effective mass of carriers. Therefore, an increase in *n* will lead to a decrease in *α*. Figure 3c gives the *α* as a function of *n* based on a single parabolic band (SPB) model and assuming a dominated scattering by acoustic phonons, which is called the Pisarenko curve. The m* of Cu_2−x_S in this work (the red line) is 6.5 m_e_. It is much higher than that of the reported data for Cu_2_S (0.5 m_e_) bulk [50]. In ref. [50], the *α* of the Cu_2_S is 280 μV K^−1^ with *n_H_* ~ 2.3 × 10^18^ cm^−3^, whereas the *α* of the present Cu_2−x_S is 271 μV K^−1^ with *n_H_* ~ 1.22 × 10^20^ cm^−3^. According to Equation (5), the Cu_2−x_S will have a much higher m*. Therefore, the Cu_2−x_S with a large m* is due to it having a high *n_H_*. In the present case, the Cu_2−x_S consisted of two phases: Cu_2_S and Cu_1.96_S. The Cu_1.96_S, which possesses more copper vacancies, would have increased the *n_H_* [28,52]. And manganese doping (y = 0.05) effectively reduced the m* to 4.2 m_e_, which is close to the value of Cu_2_S_1−x_Te_x_ [51] (m* = 4.5 m_e_). Consequently, a higher *n* and reduced m* adjusted the α to a moderate value. Additionally, the decreased m* also had an impact on *μ* according to the equation:

(6) $$\mu =\frac{q\tau }{m^{*}}$$

where *τ* is the carrier relaxation time. The decrease in m* caused the enhancement of *μ* after doping. As a result, the Cu_2−x_Mn_y_S (y = 0.05) film possessed a higher PF of ~113.3 μW m^−1^ K^−2^ at RT (~152.1 μW m^−1^ K^−2^ at 413 K), nearly twice that of the Cu_2−x_S film (58.5 μW m^−1^ K^−2^). Table 1 shows a comparison of TE performance between this work and the reported copper-sulfide-based bulks and flexible films. The PF value of the Cu_2−x_Mn_0.05_S film was superior to those of previously reported Cu_2_S-based flexible films and most bulks. However, it was lower than the PF value in ref. [21]. This may have been due to the fact that the Cu_2−x_Mn_0.05_S film was relatively porous compared to the SPS sintered bulk.

**Figure 4:**
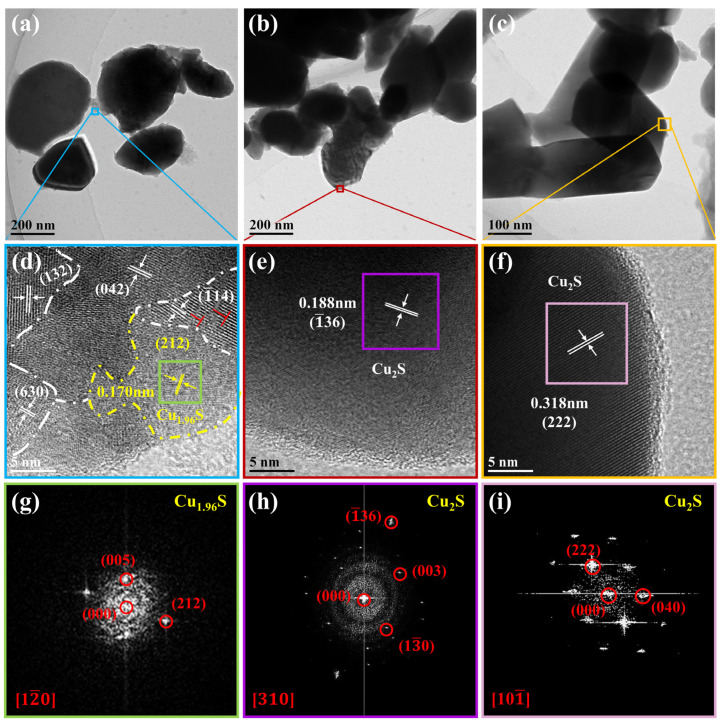
Microstructure characterization of grains scraped from the Cu_2−x_Mn_0.05_S film. (**a**–**c**) TEM images. (**d**–**f**) Enlarged images of the blue, red, and orange squares marked in (**a**–**c**), respectively. The “_┴_” in (d) denotes edge dislocation. (**g**–**i**) FFT images corresponding to the grains marked with green, purple, and pink squares in (**d**–**f**), respectively.

To further understand the internal microstructure, TEM was applied for observation of the grains scraped from the optimal film, and the result is shown in Figure 4. The grain sizes were ~20–300 nm. In the HRTEM image (Figure 4d), the measured lattice spacings of 0.305, 0.266, 0.315, and 0.194 nm are in good agreement with those of the (132), (042), (−114), and (630) planes of monoclinic Cu_2_S, respectively. And there are edge dislocations in the grains, which may be the lattice distortion caused by Mn doping. In addition, Cu_1.96_S is observed. Figure 4g shows an FFT image of the grain circled in a yellow dotted line, and the interplanar spacing is about 0.170 nm, corresponding to that of the (212) plane of Cu_1.96_S [52]. Figure 4e,f,h,i demonstrate that the lattice spacings are 0.188 and 0.318 nm, which correspond to the (−136) and (222) planes of Cu_2_S. Thus, the TEM results also confirm that the Cu_2−x_Mn_0.05_S film contained two the phases, Cu_2_S and Cu_1.96_S.

**Figure 5:**
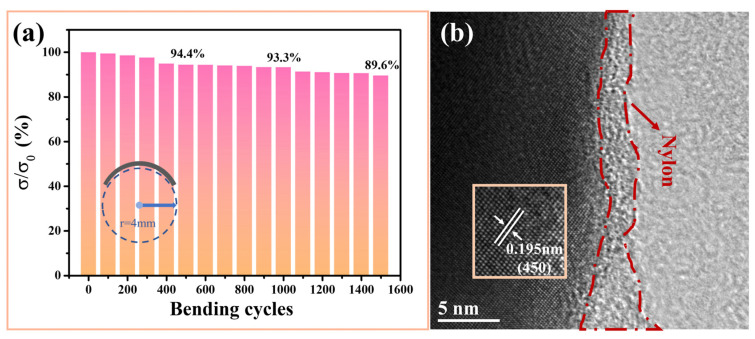
(**a**) Flexibility of the Cu_2−x_Mn_0.05_S film: The relative electrical conductivity changes with bending around a bending radius of 4 mm. (**b**) An HRTEM image of the Cu_2−x_Mn_0.05_S film showing a good combination between the film and the nylon membrane; the inset is the corresponding IFFT image.

Flexibility is also a key factor in the practical application of f-TEFs. The thickness of the Cu_2−x_Mn_0.05_S film was approximately 8.96 μm (see Supplementary Figure S7). And Figure 5a shows the corresponding flexibility test result: the *σ* maintained at 94.4%, 93.3%, and 89.6% of the original after being bent around a 4 mm-radius rod 500, 1000, and 1500 times, respectively. This is better than the flexibility of the reported Cu_2_S/PEDOT:PSS composite films [32] (the resistance rose 10% after bending 1000 cycles under a bending radius of 4 mm). The main reasons for the good flexibility are as follows: (1) the nylon membrane possessed excellent flexibility and (2) the combination between the porous film and the nylon membrane was good (see Figure 5b).

**Figure 6:**
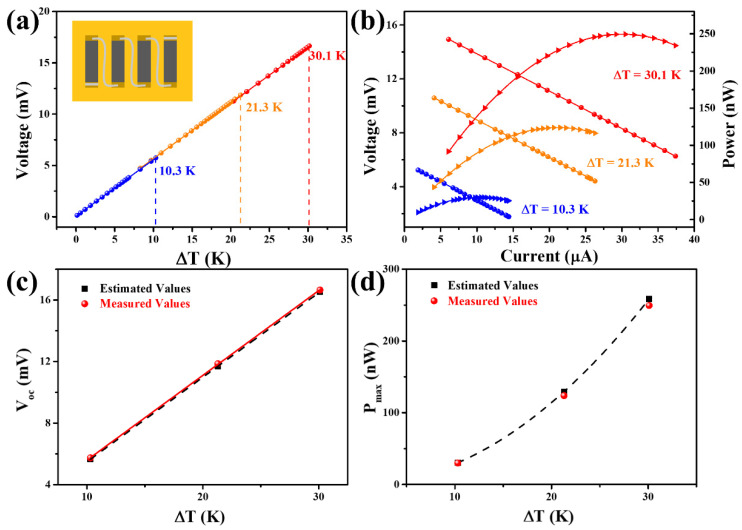
Output performance of the f-TEG assembled with the Cu_2−x_Mn_0.05_S film. (**a**) The open-circuit voltage at different Δ*T* (the inset is a schematic diagram of the four-leg f-TEG). (**b**) Output voltage and power versus current at different Δ*T*. The straight lines and curves in (**b**) correspond to V-I and P-I relations, respectively. Comparison of the estimated and measured values of the (**c**) open circuit voltage (*V_oc_*) and (**d**) maximum output power (*P_max_*) under different Δ*T*.

Figure 6a gives the variation of open-circuit voltage (*V_oc_*) with Δ*T*. When the Δ*T* values were 10.3, 21.3, and 30.1 K, the *V_oc_* values of the f-TEG were 5.74, 11.86, and 16.65 mV, respectively, which is close to the values (see Figure 6c) calculated by the equation: $V_{oc}=\left|\alpha \right|\cdot N\cdot ∆T$ (*N* is the number of f-TEG legs). Figure 6b shows the output properties of the f-TEG by adjusting the load resistance (*R_load_*) under different Δ*T*. Output voltage (*V_out_*) and output current (*I_out_*) show a negative correlation. The output power (*P_out_*) of the f-TEG can be calculated by the equation below:

(7) $$P_{out}=\frac{{V_{out}}^{2}}{R_{in}+R_{ex}}$$

where *R_in_* is the internal resistance of the f-TEG, *R_ex_* includes *R_load_* and *R_box+ammeter_* (~15.7 Ω, the internal resistance of the variable resistance box and the ammeter). *R_in_* was measured to be 264 Ω, and the resistance of the four legs (*R*_1_) was calculated by the formula $R_{1}=N\cdot l/\sigma \cdot A$ is 249 Ω (*l* and *A* are the length and cross-sectional area of one leg). Their difference comes from the contact resistance of the f-TEG. When *R_in_* was equal to *R_ex_*, the *P_out_* reached its maximum value (*P_max_*). At Δ*T* values of 10.3, 21.3, and 30.1 K, the *P_max_* values were about 29.92, 123.87, and 249.48 nW, respectively, corresponding to *R_load_* of ~251 Ω. Therefore, *R_ex_* = *R_load_* + *R_box+ammeter_* = ~251 Ω + 15.7 Ω = ~266.7 Ω, close to the *R_in_* (264 Ω). The measured *P_max_* value was close to the value estimated by the equation: $P_{max}=V_{oc}^{2}/4R_{in}\;$(see Figure 6d; more details are shown in Supplementary Table S1). The maximum power density (*PD_max_*) can be obtained by dividing *P_max_* by the total cross-sectional area of the f-TEG. It was 1.23 W m^−2^ at a Δ*T* of 30.1 K, which indicates that this f-TEG has a good potential for powering low-power consumption wearable electronics.

## 4. Conclusions

In summary, we synthesized a series of Mn-doped Cu_2−x_S powders by a green and facile hydrothermal method and successfully prepared Mn-doped Cu_2−x_S/nylon flexible films. The doping of Mn increased the *n* and *μ*, which ultimately improved the *σ* of the Cu_2−x_Mn_y_S films. The optimal film (Cu_2−x_Mn_0.05_S) exhibited a high PF of 113.3 μW m^−1^ K^−2^ at RT, with an enhanced *σ* of 60.1 S cm^−1^ and a proper *α* of 137.3 μV K^−1^. At the same time, this film possessed good flexibility: the *σ* was maintained at ~93.3% after bending 1000 times around a rod with a radius of 4 mm. An assembled four-leg f-TEG produced a maximum power of 249.48 nW (corresponding power density ~1.23 W m^−2^) at a Δ*T* of 30.1 K. Our results indicate that Mn doping is an effective and convenient to improve the electrical properties of Cu_2−x_S and provides the possibility of fabricating low-cost and high-flexibility copper-sulfide-based films for powering wearable devices.

## Figures and Tables

**Table 1:** TE performance of the reported copper-sulfide-based bulks and flexible films.

Materials	PF (μW m^−1^K^−2^)	Temperature (K)	Method	Ref. No
Cu_2−x_S	56	300	Solvothermal and HP	[52]
Cu_2−x_S	75	300	Melting and SPS	[53]
Cu_1.98−2x_Mn_x_S_0.985_Se_0.015_	100	325	Melting and SPS	[47]
Cu_2_S_1−x_Se_x_	122	325	Ball milling and SPS	[24]
Micro/nano Cu_2−x_S	250	320	Hydrothermal and SPS	[21]
Cu_2_S_1−x_Te_x_	145	325	Ball milling and SPS	[54]
Cu_2_S hybrid films	20	393	Screen printing	[31]
Cu_2_S/PEDOT:PSS films	56	393	Vacuum filtration	[32]
Cu_2−x_Mn_y_S/nylon films	113	300	Hydrothermal and HP	This work
	150	393		

Note: The data related to copper-sulfide-based bulks are estimated from the relevant reported graphs.

## Data Availability

Data are contained within the article and Supplementary Materials.

## Supplementary Materials

The following supporting information can be downloaded at: https://www.mdpi.com/article/10.3390/ma16227159/s1. Figure S1: Schematic diagram of the preparation process of the Cu2−xMnyS film; Figure S2: A schematic diagram for the output performance measurement of the TE generator; Figure S3: XRD patterns of the Cu2−xMnyS powders with varying doping content of Mn; Figure S4: SEM images of Cu2−xMnyS powders. (a) y = 0.01, (b) y = 0.03, (c) y = 0.05, (d) y = 0.07; Figure S5: SEM images of the Cu2−xMnyS films. (a) y = 0.01, (b) y = 0.03, (c) y = 0.05, (d) y = 0.07; Figure S6: XPS spectra of the Cu2−xMn0.05S powders. (a) survey scan. (b–d) high-resolution scans for Cu 2p, S 2p and Mn 2p, respectively; Figure S7: Cross-sectional SEM image of the Cu2−xMn0.05S film; Table S1. The measured and estimated values of the open-circuit voltage (Voc) and maximum output power (Pmax) of the f-TEG at different ΔT.
