# Supplementary material for: Largely Enhanced Thermoelectric Power Factor of Flexible Cu2−xS Film by Doping Mn
Source: Materials (Basel). 2023 Nov 14;16(22):7159. doi: 10.3390/ma16227159 (PMC10672275; doi:10.3390/ma16227159)
Supplement: Supplementary file 1 [file materials-16-07159-s001.zip › materials-2705017-SI.pdf]

Supplementary Material

Largely enhanced thermoelectric power factor of flexible $Cu_{2-x}S$ film by doping Mn

Xinru Zuo, Xiaowen Han, Yiming Lu, Ying Liu, Zixing Wang, Jiajia Li and Kefeng Cai*

Key Laboratory of Advanced Civil Engineering Materials of Ministry of Education, Shanghai Key Laboratory of Development and Application for Metal-Functional Materials, School of Materials Science & Engineering, Tongji University, Shanghai, 201804, China

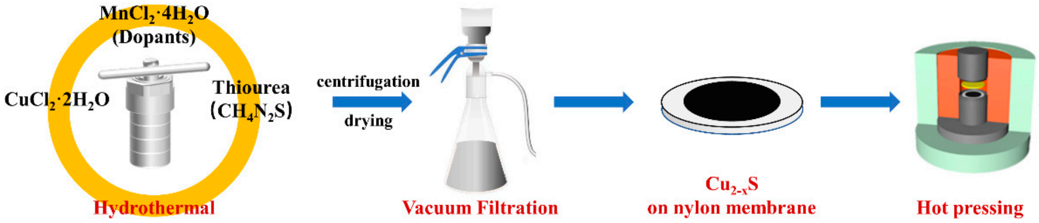

Fig. S1. Schematic diagram of the preparation process of the $Cu_{2-x}Mn_yS$ film.

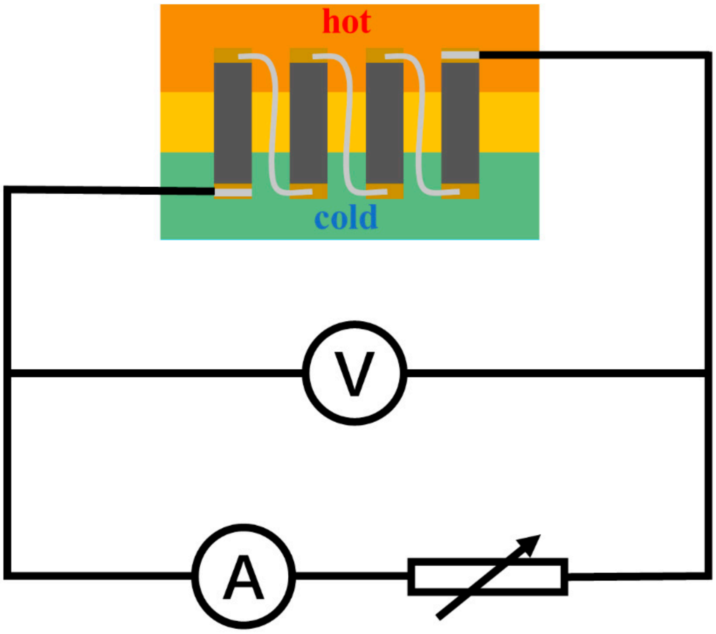

Fig. S2. A schematic diagram for the output performance measurement of the TE generator.

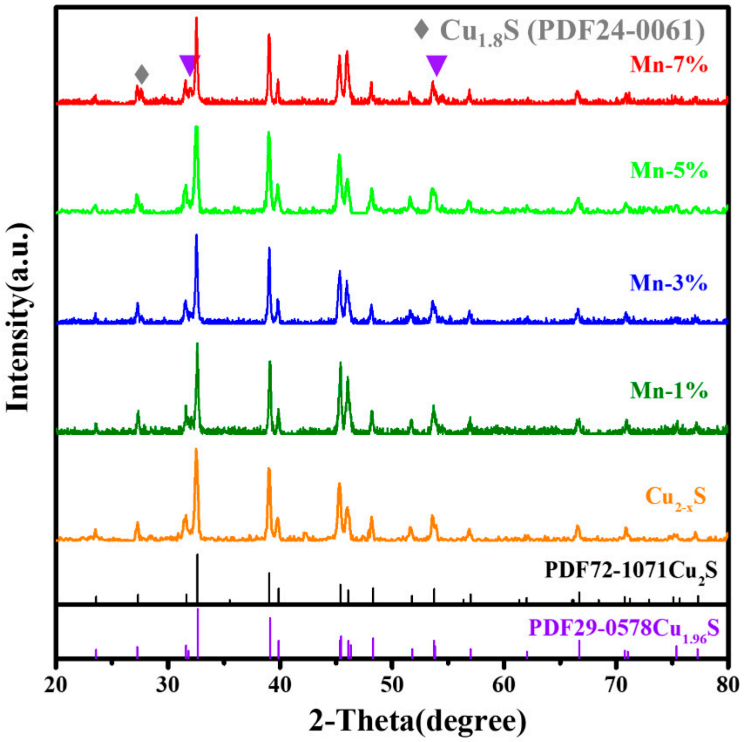

Fig. S3. XRD patterns of the $Cu_{2-x}Mn_yS$ powders with varying doping content of Mn.

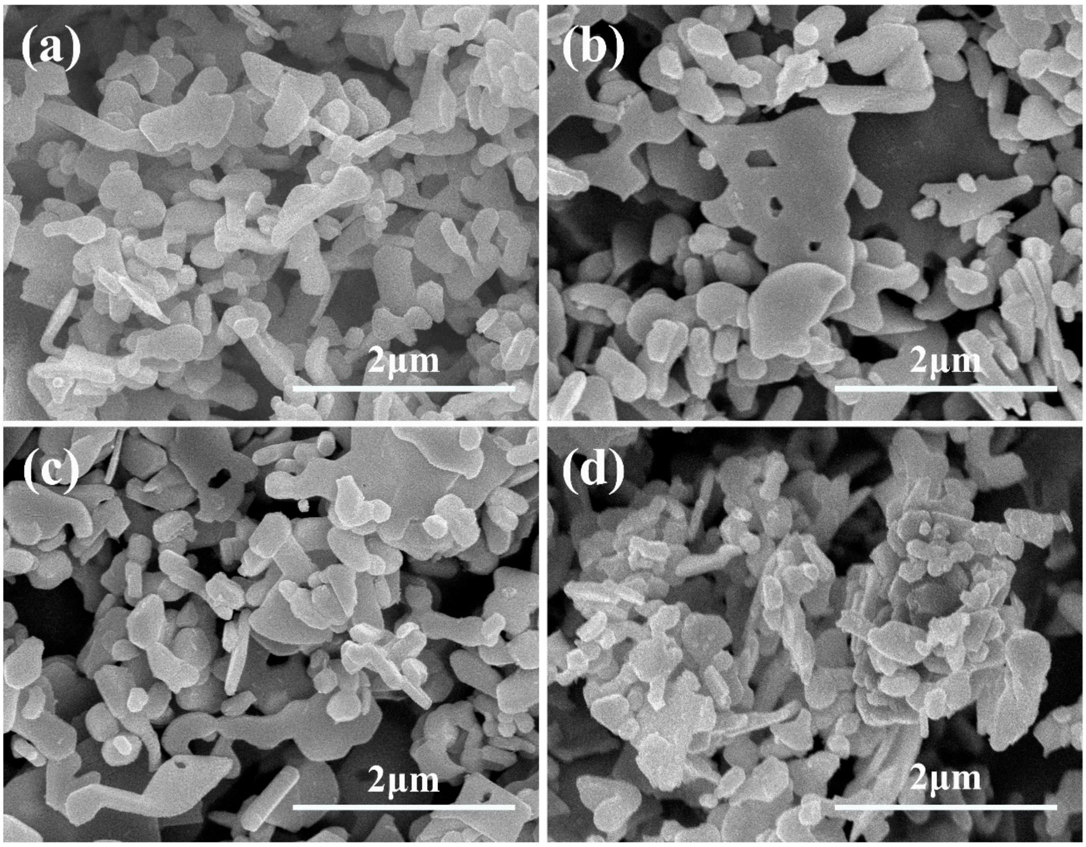

Fig. S4. SEM images of $Cu_{2-x}Mn_yS$ powders. (a) y=0.01, (b) y=0.03, (c) y= 0.05, (d) y=0.07.

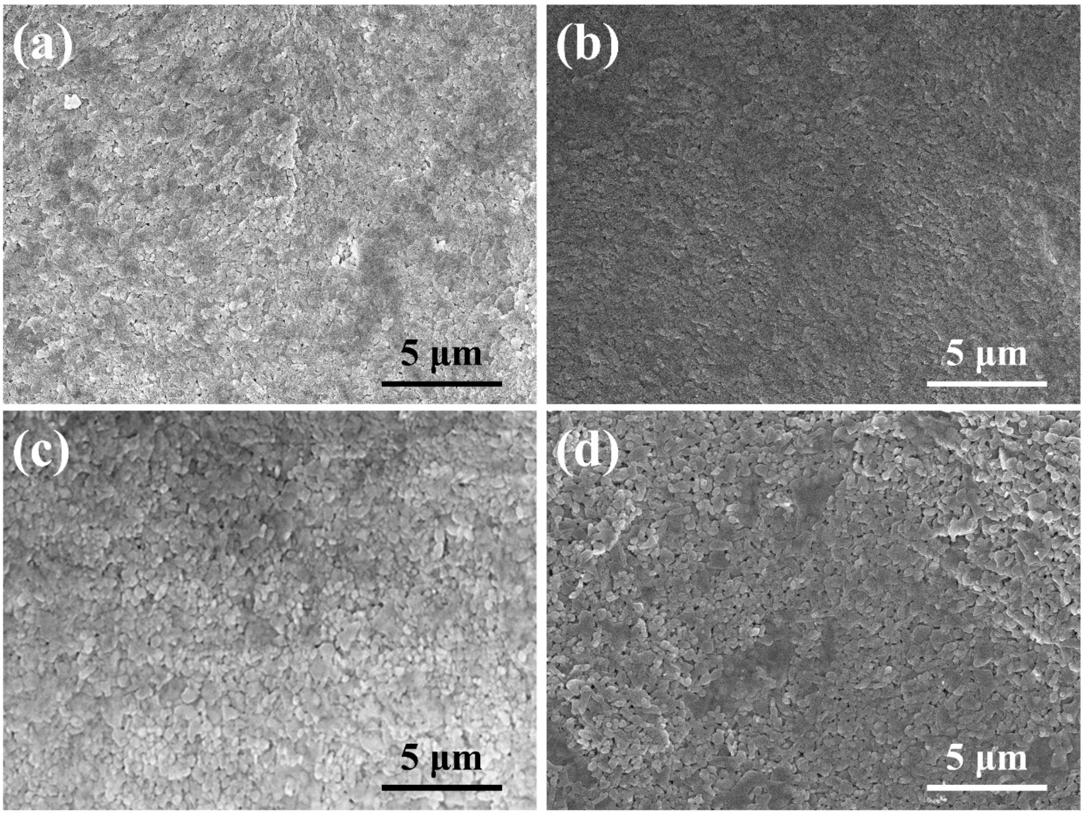

Fig. S5. SEM images of the $Cu_{2-x}Mn_yS$ films. (a) y=0.01, (b) y=0.03, (c) y= 0.05, (d) y=0.07.

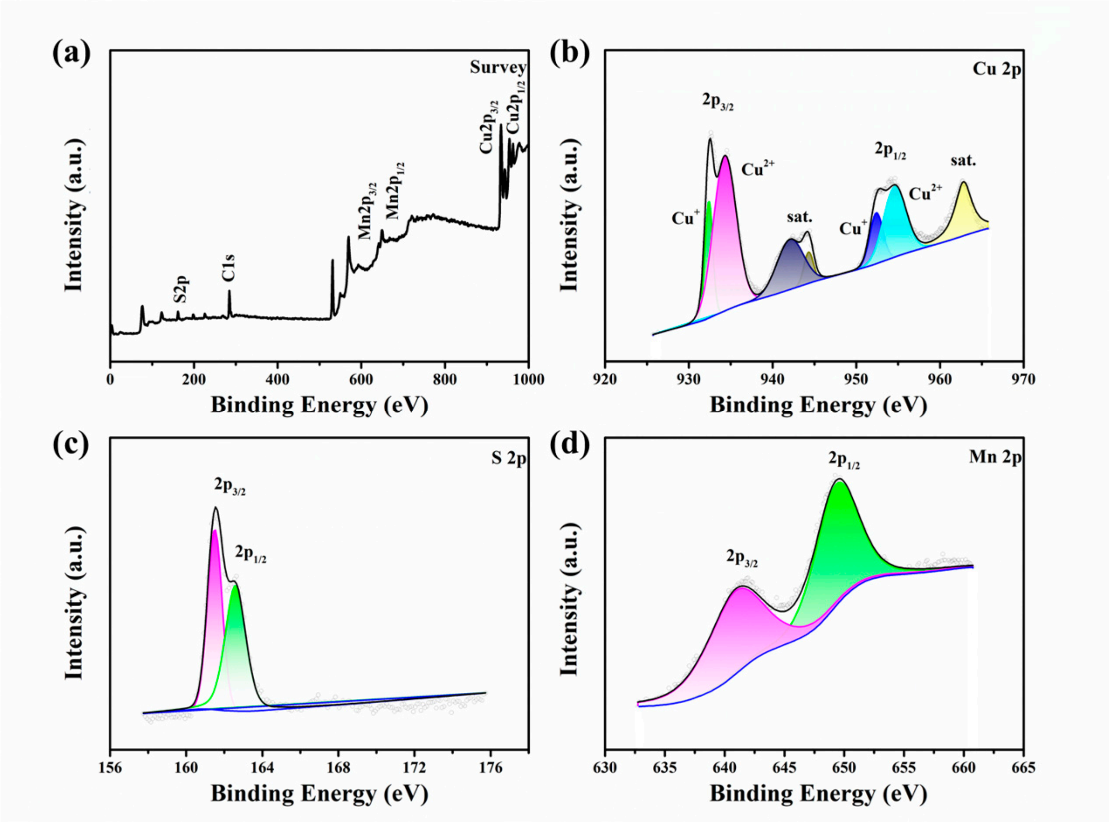

Fig. S6. XPS spectra of the $Cu_{2-x}Mn_{0.05}S$ powders. (a) survey scan. (b-d) high-resolution scans for Cu 2p, S 2p and Mn 2p, respectively.

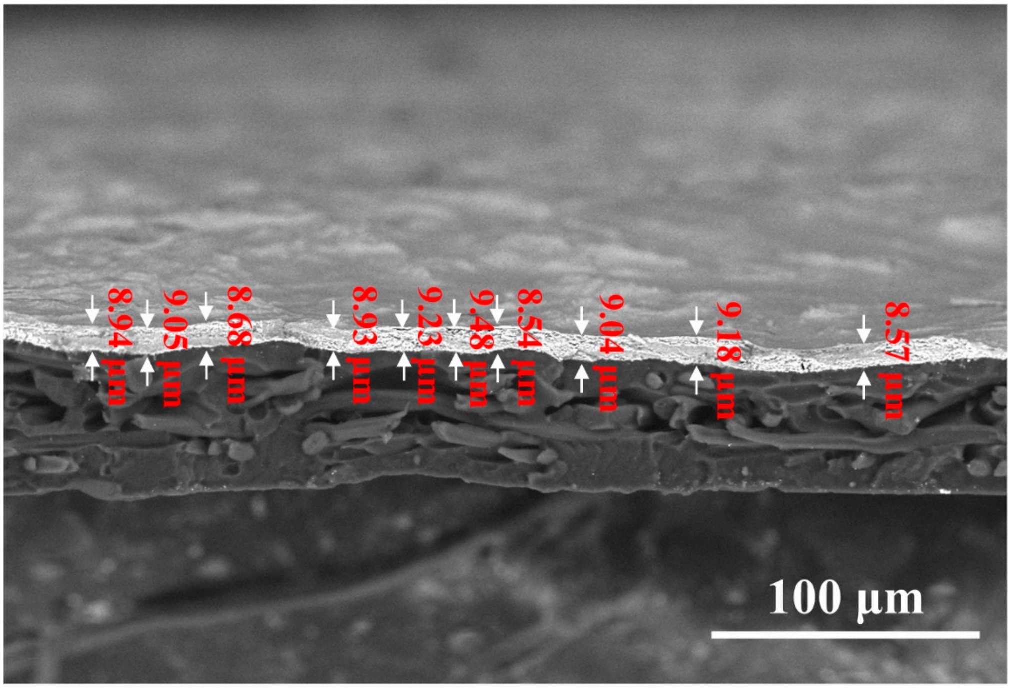

Fig. S7. Cross-sectional SEM image of the $Cu_{2-x}Mn_{0.05}S$ film.

Table S1. The measured and estimated values of the open-circuit voltage (V_{oc}) and maximum output power (P_{max}) of the f-TEG at different ΔT.

ΔT (K)	V_{oc} (mV)		P_{max} (nW)	
	Measured values	Estimated values	Measured values	Estimated values
10.3	5.74	5.65	29.92	30.2
21.3	11.86	11.69	123.87	129.4
30.1	16.65	16.53	249.48	258.7
